# Factors in oral-related quality of life of betel quid users receiving oral mucosal screening: a cross‑sectional study in Taiwan

**DOI:** 10.1186/s12903-023-02800-x

**Published:** 2023-02-12

**Authors:** Su-Erh Chiu, Chung-Jan Kang, Shu-Ching Chen

**Affiliations:** 1 Department of Nursing, Linkou Chang Gung Memorial Hospital, Taoyuan, Taiwan; 2 Department of Otorhinolaryngology, Head and Neck Surgery, Linkou Chang Gung Memorial Hospital, Taoyuan, Taiwan; 3 Department of Medicine, College of Medicine, Chang Gung University, Taoyuan, Taiwan; 4 School of Nursing and Geriatric and Long-Term Care Research Center, College of Nursing, Chang Gung University of Science and Technology, 261, Wen-Hua 1st Road, Guishan, Taoyuan, 333 Taiwan; 5 Department of Radiation Oncology and Proton and Radiation Therapy Center, Linkou Chang Gung Memorial Hospital, Taoyuan, Taiwan; 6 School of Nursing, College of Medicine, , Chang Gung University, Taoyuan, Taiwan

**Keywords:** Betel quid, Cancer screening, Oral mucosal screening, Oral-related quality of life

## Abstract

**Background:**

Betel quid (BQ) chewing is associated with poor oral hygiene, psychological impairment, and acute and long-term addictive effects, resulting in worse oral-related quality of life (OHRQoL). The purpose of this study was to characterize the factors associated with OHRQoL among BQ users receiving oral mucosal screening.

**Methods:**

A cross-sectional study was conducted. Data were collected by random sampling of BQ users who visited outpatient departments receiving oral mucosal screening in a medical center Taiwan. The oral health assessment tool, the state anxiety inventory, the betel quid dependence scale, and the oral health impact profile were used to measure oral health status, anxiety, BQ dependence, and OHRQoL, respectively. Pearson’s product-moment coefficient was used to examine the relationship between OHRQoL and the selected independent variables. Independent-samples *t-*test was used to compare OHRQoL by annual family income, the presence of chronic disease, and BQ dependence. Hierarchical multiple linear regression analysis was used to identify factors associated with OHRQoL.

**Results:**

A total of 175 BQ users were surveyed. Factors associated with OHRQoL included oral health status, anxiety, and BQ dependence. BQ users reporting low oral health status, greater anxiety, and more BQ dependence were more likely to have worse OHRQoL.

**Conclusions:**

Poor oral health status, anxiety, and BQ dependence negatively impact on OHRQoL among patients with BQ use receiving oral mucosal screening.

## Background

Betel quid (BQ) chewing is a global health issue with high prevalence in South Asia, East Africa, and the Pacific region [1]. In Taiwan, although the prevalence is decreasing, the health consequences of BQ chewing remain problematic [2]. BQ chewing causes poor oral hygiene, psychological impairment, acute and long-term addictive effects [3], worse oral-related quality of life (OHRQoL), and results in metabolic syndrome, diabetes [3], precancerous oral lesions, and oral cavity cancer [4].

BQ use causes quid-induced lesions such as color changes, rough linen-like texture, and para-keratinized epithelium [5]. Oral mucosal lesions can develop into premalignant lesions; oral mucosal screening allows early identification of suspicious oral mucosal lesions [6]. Physicians use gloved hands and a hand-held light to check the mucosa of the oral cavity in order to detect oral mucosal abnormalities [7]. Early oral mucosal screening can detect oral precursor lesions in a high-risk population, decreasing oral cavity cancer mortality by up to 43% [7]. In Taiwan, people recommended to receive a biennial oral mucosal screening include those aged 30 years and above with a BQ chewing or smoking habit or those aged 18–29 years with a BQ chewing habit [7]. Approximately 450,000 people received oral mucosal screening annually, although attendance dropped 18% in 2022 due to the outbreak of the COVID-19 pandemic. People wearing face masks experience an additional barrier to oral mucosal screening [8, 9].

People with BQ use often neglect oral hygiene due to unhealthy dental care habits and negative attitudes toward oral health [10]. Studies show that, compared to non-users, BQ users have increased periodontal inflammatory conditions such as bleeding, high plaque index, and gingivitis [11, 12], BQ users often experience emotional distress, such as anxiety [13], depression [13], and distress related to betel quid dependence (BQD), such as tolerance, cravings, substance-seeking behavior, and withdrawal symptoms [14–16]. These problems may threaten health behaviors related to personal hygiene, health promotion, and overall well-being.

OHRQoL refers to personal perceived oral health condition and is a multidimensional measure composed of physical, social, and psychological dimensions. Lower OHRQoL may negatively impact eating, speaking, daily life, and social interaction [17]. Lower OHRQoL is also associated with younger age [18], being man [19], lower educational level [20], less social economic status [21], more dental caries, more severe periodontal disease, poor oral hygiene care [22], poor health status [23], and psychological impairment [24]. The biopsychosocial model for OHRQoL proposed by Wilson and Cleary [25] and Ferrans [26] posits that unfavorable personal characteristics, environmental factors, physical, social, and psychosocial functioning generate negative health perceptions and result in worse OHRQoL. According to this model, we propose that OHRQoL is affected by personal characteristics (educational level), environmental factors (annual family income), physical (chronic disease and oral health status), social, and psychosocial functioning (anxiety and BQD). Of the few studies that have explored this topic, most have researched populations in Western Asia [10, 11], northern India [24], South Asia [12], older adults [18, 19, 21], and patients with diabetes [23]. Therefore, the aims of this study are, in BQ users receiving oral mucosal screening, (1) to investigate the levels of oral health, anxiety, BQD, and OHRQoL; and (2) to examine the factors (e.g., educational level, annual family income, chronic disease, oral health status, anxiety, and BQD) associated with OHRQoL.

## Methods

### Design and sample

This was a cross-sectional survey study. Participants were selected as study subjects using random sampling from October 2019 to September 2021 while visiting the oral mucosal screening of the otorhinolaryngology outpatient department of a leading medical center in northern Taiwan. The inclusion criteria were as follows: (1) age 20 years or above; (2) having a BQ chewing habit and receiving oral mucosal screening; and (3) able to communicate in Mandarin or Taiwanese.

The steps of random sampling were as follows: (1) define the population: the eligible population included all 2000 BQ users who received oral mucosal screening at Chang Gung Memorial Hospital during the study period; (2) decide on sample size: the sample included 175 of BQ users who received oral mucosal screening in an average year. This fraction of the overall population of BQ users has enough sample power to represent the population of Taiwan; (3) random selection: this institute randomly selects the addresses of 2000 BQ users per year for testing. Each address has approximately a 1-in-8 chance of being selected. Of those who received screening, 180 were randomly selected for inclusion; and (4) data collection: 175 subjects filled out the self-report survey.

### Measures

#### Oral health assessment tool (OHAT)

Oral health status was measured using the Chinese version of the oral health assessment tool (OHAT) [27], developed by Chalmers et al. [28] OHAT is composed of 12 questions concerning the lips, tongue, gums, saliva, natural teeth, dentures, oral cleanliness, and dental pain. Responses are scored on a scale of 0–2; summed OHAT scores range 0–16 (≤ 3 as healthy, ≥ 4 as unhealthy), with higher scores indicates worse oral health and more oral and dental problems [28]. In this study, the Cronbach’s alpha value for the OHAT was 0.90.

#### State anxiety inventory (SAI)

The state anxiety inventory (SAI), used to assess patients’ anxiety related to a particular situation, is a 20-item measure; each response is made on a 1–4 scale and summary scores range from 20 to 80, with higher scores indicating higher levels of anxiety [29]. The summary scores are classified into two leveles of anxiety: scores > 44 indicate a high level of anxiety and scores ≤ 44 represent mild-to-moderate anxiety [30]. The SAI was translated into Chinese, and previous studies reported satisfactory psychometric properties [31, 32]. For the present study, the Cronbach’s alpha was 0.90.

#### Betel quid dependence scale (BQDS)

BQ-related withdrawal symptoms were measured with the Betel Quid Dependence Scale (BQDS) for BQ users, developed from the Diagnostic and Statistical Manual of Mental Disorders, 4th Edition for substance dependence [33]. Its 16 items belong to three domains of BQD: physical and psychological urgent need (7 items), increasing dose (5 items), and maladaptive use (4 items) [34]. Each item is assigned a score 0 (no) or 1 (yes), with the total possible score ranging from 0 to 16, higher scores indicating higher BQD. Scores of 4 or greater indicate BQD [21]. We used the Chinese version of the BQDS, which has acceptable reliability and validity and optimal sensitivity and specificity [33]. In this study, the Cronbach’s α value was 0.92.

#### Oral health impact profile (OHIP)

OHRQoL was measured using the Chinese version of the oral health impact profile (OHIP), developed by Slade [35] and translated and verified by Kuo et al. [36] It consists of 14 items: 2 each for functional limitation, physical discomfort, psychological discomfort, physical disability, psychological disability, social disability, and handicap. Answers are provided for each item using a 5-point Likert scale ranging from 0 (never) to 4 (very often). The total score ranges from 0 to 56, with a higher score indicating lower levels of function or poorer oral health status. In our study, the Cronbach’s α value for this instrument was 0.90.

#### Demographic and betel quid (BQ)-related characteristics form

Subject-related characteristics included age, sex, marital status, education level, type of occupation, religion, and annual family income (in New Taiwan dollars, NT$). Income was divided into NT$500,000 or below versus higher. BQ-related records included smoking status, alcohol drinking status, duration of BQ chewing, motivation for oral mucosal screening, and clinical presentation at oral mucosal screening.

### Procedure for data collection

Potential eligible subjects were selected from physician referrals or those who actively made an appointment for oral mucosal screening and who were met the criteria. A research nurse approached each potential subject and provided a set of questionnaires to be completed by self-report before the patient visited the otorhinolaryngology physician and received an oral mucosal screening. The content of the questionnaires was set at a fifth-grade reading level without medical terminology. All of our participants were literate and able to read and write independently; when necessary, a research nurse helped by reading out each item of the questionnaires.

### Statistical analysis

Before data analysis, we examined the data that showed a normal distribution. Parametric statistical analysis was used. We analyzed data by using SAS for Windows, version 9.1 (SAS Institute, Inc., Cary, NC). Descriptive statistics were used to analyze demographic and clinical characteristics, oral health status, anxiety, BQD, and OHRQoL. Pearson’s product-moment coefficient was used to examine the relationships between OHRQoL (dependent variable) and the selected independent variables. The independent-samples *t-*test was used to compare levels of OHRQoL by annual family income, presence of chronic disease, and BQD. Considering that the sample size of females (n = 2) was less than 30, we used non-parametric testing for inferential statistics [37]. The Mann–Whitney U test was used to compare levels of OHRQoL by sex. Hierarchical multiple linear regression analysis was used to assess the variables associated with OHRQoL. The independent variables found to be statistically significant in Pearson’s product moment coefficient analysis, independent-samples *t-*test, or Mann–Whitney U test were included into the hierarchical multiple linear regression analysis (oral health status, anxiety, BQD, and annual family income).

Statistical power was calculated using the G*Power 3.1.9 program [38, 39] for hierarchical multiple linear regression [40]. For this study, a total of 175 subjects was found to reach a statistical power of 0.80 and effect size of 0.35 [40] for 3 independent variables in step 1 analysis, and 4 independent variables in step 2 analysis, with a significance level (α) of 0.05.

### Ethical considerations

Ethical permission was obtained from the Ethics Committee of Chang Gung Medical Foundation in Taiwan (Number: 201900801B0). All procedures were performed in accordance with the Declaration of Helsinki. Written consent was obtained from all participants before recruitment.

## Results

### Demographic and clinical characteristics

Of 180 eligible BQ users approached, 175 BQ users participated in this research, for a response rate of 97.2%. The mean age was 51.27 years (standard deviation [SD] = 10.40; range 21–74 years). Most of the BQ users were male (98.9%). Of the total sample, 61.1% were living alone, 49.1% were educated at the senior-high level, 37.7% were skilled workers, 65.7% reported their religion as Buddhism or Taoism, and 52.0% had an average family annual income less than NT$500,000. Most BQ users reported being current smokers (78.9%), current alcohol drinkers (69.7%), had chewed BQ for more than 10 years with chewing more than 20 pieces per day (n = 57, 32.6%), received oral mucosal screening by doctor referral (n = 125, 71.4%), and had a clinical presentation of normal (n = 156.1, 89.1%) (Table 1).

**Table 1:** Demographic and clinical characteristics of patients (*n* = 175)

Variable	Number (%)
Sex	
Male	173 (98.9)
Female	2 (1.1)
Marital status	
Living alone	107 (61.1)
Living with a partner	68 (38.9)
Educational level	
Literacy	3 (1.7)
Elementary	17 (9.7)
Junior high	41 (23.4)
Senior high	86 (49.1)
College and above	28 (16.0)
Type of occupation	
Unemployed	17 (9.7)
Unskilled/ semi-skilled worker	15 (8.6)
Skilled worker	66 (37.7)
Clerk, shop owner, farm owner	35 (20.0)
Semi-professional	22 (12.6)
Professional	8 (4.6)
Other	12 (6.8)
Religion	
None	48 (27.4)
Buddhism/Taoism	115 (65.7)
Christianity/Catholicism	10 (5.7)
Other	2 (1.2)
Annual family income (NT$)	
< 500,000	91 (52.0)
≥ 500,000	84 (48.0)
Smoking	
Nil/ex-smoker	37 (21.1)
Current smoker	138 (78.9)
Drinking	
Nil/ex-drinker	53 (30.3)
Current drinker	122 (69.7)
Time since chewing betel nut	
< 10 years, < 20 amount/day	34 (19.4)
< 10 years, > 20 amount/day	29 (16.6)
> 10 years, < 20 amount/day	55 (31.4)
> 10 years, > 20 amount/day	57 (32.6)
Motivation for oral mucosal screening	
Active screening	31 (17.7)
Family members advice	19 (10.9)
Doctor referral	125 (71.4)
Clinical presentation	
Normal	156 (89.1)
Suspected oral cavity cancer	9 (5.1)
Unknown oral mass	1 (0.6)
Erythroplakia (red)	2 (1.1)
Erythroleukoplakia (red and white)	1 (0.6)
Thick homogeneous leukoplakia	3 (1.7)
Thin homogeneous leukoplakia	1 (0.6)
Oral ulcer	2 (1.1)

*SD* standard deviation, *NT* New Taiwan Dollars

### Degree of oral health status, anxiety, betel quid dependency (BQD), and oral-related quality of life (OHRQoL)

The mean score for oral health status was 5.61 (SD = 3.08). Based on the OHAT classification [28], 28.6% (n = 50) of BQ users were healthy and 71.4% (n = 125) were unhealthy. The mean score for state anxiety was 51.51 (SD = 10.78). According to the SAI classification [30], 21.19% (n = 37) of BQ users had mild-to-moderate anxiety, and 78.9% (n = 138) had a high level of anxiety. The mean score for BQDS was 7.78 (SD = 4.04). The mean BQDS subscales were: 3.72 (SD = 2.13) for physical and psychological urgent need, 2.68 (SD = 1.51) for increasing dose, and 1.38 (SD = 1.33) for maladaptive use dose. According to the standard BQD classification [31], 84.6% (n = 148) of the BQ users had BQD and 15.4% (n = 27) did not. The mean score for overall OHRQoL was 27.43 (SD = 8.78). Mean scores for the subscales were as follows: functional limitation, 2.82 (SD = 1.56); physical pain, 4.44 (SD = 1.89); psychological discomfort 4.67 (SD = 1.71); physical disability, 3.63 (SD = 1.54); psychological disability, 4.43 (SD = 1.76); social disability, 4.06 (SD = 1.90), and handicap, 3.38 (SD = 1.35) (Table 2).

**Table 2:** Degree of oral health status, anxiety, betel quid dependency, and oral-related quality of life (*n* = 175)

Variable	Mean (SD)	Actual scoring range	Theoretical scoring range	Number (%)
Oral health status (OHAT)	5.61(3.08)	0–13	0–16	
Healthy				50 (28.6)
Unhealthy				125 (71.4)
State anxiety (SAI)	51.51 (10.78)	20–75	0–80	
Mild-to-moderate anxiety				37 (21.1)
High anxiety				138 (78.9)
Betel quid dependence syndrome (BQDS)	7.78 (4.04)	0–16	0–16	
Physical and psychological urgent need (BQDS)	3.72 (2.13)	0–7	0–7	
Increasing dose (BQDS)	2.68 (1.51)	0–5	0–5	
Maladaptive use (BQDS)	1.38 (1.33)	0–4	0–4	
Without BQD				27 (15.4)
With BQD				148 (84.6)
Oral-related quality of life (OHIP)	27.43 (8.78)	14–57	0–70	
Functional limitation (OHIP)	2.82 (1.56)	2–10	2–10	
Physical pain (OHIP)	4.44 (1.89)	2–10	2–10	
Psychological discomfort (OHIP)	4.67 (1.71)	2–10	2–10	
Physical disability (OHIP)	3.63 (1.54)	2–10	2–10	
Psychological disability (OHIP)	4.43 (1.76)	2–10	2–10	
Social disability (OHIP)	4.06 (1.90)	2–10	2–10	
Handicap (OHIP)	3.38 (1.35)	2–10	2–10	

*OHAT* oral health assessment tool; higher scores indicate more oral and dental problems; based on the OHAT scores, were divided into two categories of oral health, the OHAT score ≤ 3 as healthy, and the OHAT score ≥ 4 as unhealthy

*SAI* state anxiety inventory; higher scores indicate higher levels of anxiety; based on the SAI scores, were divided into two categories of anxiety, the SAI score > 44 indicate high level of anxiety and scores ≤ 44 represent mild-to-moderate anxiety

*BQDS* betel quid dependence scale; higher scores indicate higher betel quid dependence; based on the BQDS scores, were divided into two categories of betel quid dependence syndrome, the BQDS score < 4 as not BQDS cases, and the BQDS score ≥ 4 as BQDS cases

*OHIP* oral health impact profile; higher scores indicate lower levels of function or poor oral health status

*SD* standard deviation

### Correlation of OHRQoL with selected independent variables

The factors that correlated significantly with OHRQoL were oral health status (*r* = 0.258, *p* < 0.01) and anxiety (*r* = 0.302, *p* < 0.01). These factors were included as independent variables in the hierarchical regression analysis. OHRQoL was negatively correlated with age (*r* = − 0.122, *p* > 0.05) and educational level (*r* = − 0.082, *p* > 0.05), but the results were not statistically significant (Table 3).

**Table 3:** Correlations of oral-related quality of life with selected independent variables (*n* = 175)

Variable	1	2	3	4	5
(1) Age	1.000				
(2) Education level (year)	− 0.438**	1.000			
(3) Oral health status	0.131	− 0.087	1.000		
(4) Anxiety	− 0.073	− 0.118	0.015	1.000	
(5) Oral-related quality of life	− 0.122	− 0.082	0.258**	0.302**	1.000

^*^p < 0.05; **p < 0.01

### Differences in oral-related quality of life by sex, annual family income, presence of chronic disease, and betel quid dependence (BQD)

Of the 175 BQ users studied, 173 were male and 2 were female. Independent-samples *t*-test analysis was used to examine the differences in OHRQoL between the two groups. Female BQ users had higher level of OHRQoL than male BQ users, but the difference was not statistically significant (*z* = 0.099, *p* > 0.05).

Of the 175 BQ users studied, 91 BQ users had an average family annual income less than NT$500,000, and 84 BQ users had an average family annual income of NT$500,000 or above. Independent-samples t-test examined the differences in OHRQoL between the two groups. BQ users with an average family annual income of NT$500,000 or above had statistically significantly higher levels of OHRQoL than those with a lower average family annual income (*t* = − 4.941, *p* < 0.01).

Of the 175 BQ users studied, 80 had chronic disease, and 95 did not. Independent-samples t-test was used to examine the differences in OHRQoL between the two groups. The BQ users who had chronic disease had higher levels of OHRQoL than those BQ users who did not have chronic disease, but the difference was not statistically significant (*t* = − 1.849, *p* > 0.05).

Of the 175 BQ users studied, 148 had BQD and 27 did not. Independent-samples *t*-test was used to examine the differences in OHRQoL between the two groups. BQ users with BQD had statistically significantly higher levels of OHRQoL than those without BQD (*t* = − 3.958, *p* < 0.01) (Table 4).

**Table 4:** Differences in oral-related quality of life by sex, annual family income, status of chronic disease, and betel quid dependence (*n* = 175)

Variable	Mean	SD	*t/z*	*p*
Sex			0.099	0.107^a^
Male (*n* = 173)	27.35	3.54		
Female (*n* = 2)	34.50	8.79		
Annual family income (NT$)			− 4.941	0.001
< 500,000 (*n* = 91)	24.23	7.16		
≥ 500,000 (*n* = 84)	30.38	9.12		
Chronic disease			− 1.849	0.066
No (*n* = 95)	26.28	7.49		
Yes (*n* = 80)	28.79	9.97		
Betel quid dependence (BQDS)			− 3.958	0.001^a^
Without BQD (*n* = 27)	22.11	7.96		
With BQD (*n* = 148)	28.40	8.59		

^a^Mann–Whitney U test

### Hierarchical multiple regression analysis of OHRQoL in BQ users

Hierarchical multiple regression models were employed to identify factors associated with OHRQoL among BQ users receiving oral mucosal screening. In model 1, oral health status, anxiety, and BQD significantly contributed to the regression model (*F* = 12.355, *p* < 0.01) and accounted for 17.8% of the variation in OHRQoL. In model 2, the results showed that oral health status, anxiety, and BQD significantly contributed to the regression model (*F* = 9.216, *p* < 0.01). R^2^ did not change, representing that oral health status, anxiety, and BQD specially contributed to explain the variance in OHRQoL among BQ users. When added to the model, annual family income (*p* > 0.05) did not significantly explain the variance in OHRQoL (Table 5).

**Table 5:** Hierarchical multiple regression analysis of oral-related quality of life in BQ users (*n* = 175)

Variable	Model 1			Model 2		
	β	t	p	β	t	p
Oral health status	0.694	3.509	0.001	0.693	3.493	0.001
Anxiety	0.198	3.300	0.001	0.198	3.277	0.001
Betel quid dependence	3.915	2.190	0.030	3.905	2.175	0.031
Annual family income				0.141	0.112	0.911
F	12.355		0.001	9.216		0.001
R^2^	0.178			0.178		
ΔR^2^	0.178			0.000		

^*^p < 0.05; **p < 0.01; ***p < 0.001

## Discussion

In this study, the rate of BQ users receiving oral mucosal screening who had an unhealthy oral health status (71.4%) was similar to that of previous studies [8, 41], which also found a higher prevalence of dental caries and oral candida in BQ users than in non-users. The explanation may be that BQ chewing causes vasoconstriction and nitrosation due to nicotine and alkaloids, which also serve as nutrients for oral bacteria and facilitate the development of dental caries [42]. To improve oral health and oral hygiene, health care providers should assess the oral care habits of patients and motivate them to stop using BQ. Therefore, community-based oral care education and continuing BQ cessation programs are warranted, particularly efforts targeting BQ users.

We observed that 84.6% of our BQ users had BQD, a result similar to a previous report of a prevalence of 47.9–99.3% [43], but much higher than the 39.3% reported by Lee et al. The discrepancy between our study and published research may derive from diversity in characteristics between cohorts. All of our BQ users were current BQ chewers and most had chewed BQ for more than 10 years; in contrast, only 10.7% of the cohort of Lee et al. [16] were current BQ users. Healthcare providers should assess whether the duration and dosage of BQ chewing is related to BQD and whether it may negatively affect oral mucosal screening in their patients.

The present study demonstrated that BQ users with more anxiety and BQD were more likely to have worse OHRQoL. However, nearly three-fourths of our subjects had a high level of anxiety. Findings from this current study reflect the fact that arecoline, the main ingredient in BQ, is a nicotine-like psychoactive, which generates alertness, euphoria, elation, and relaxation, leading users to BQD. Arecoline dependence withdrawal symptoms include irritability, anxiety, insomnia, tiredness, and difficulty concentrating [44]. In Taiwan, most BQ users are blue collar laborers [45, 46], as were our study subjects. Hence, health professionals should educate such populations to perform regular physical exercise to overcome stress, motivate them to quit BQ chewing, and encourage them to regularly undergo oral mucosal screening.

In our cohort, BQ users reported worse OHRQoL related to poorer oral health status. This finding is similar to those of previous studies [47, 48] and agree with Locker’s oral health model [49]. This result might reflect poor health literacy, low self-efficacy, or insufficient knowledge of disease prevention among these patients with BQ use. Therefore, education in oral care and in avoiding hazardous substances, and providing psychological support will help relieve physical and psychological discomfort in these patients and promote oral hygiene.

In the present study, only 17.7% of BQ users actively sought out screening; the rest were screened on the advice of a family member (10.9%) or were referred to screening by a doctor (71.4%), which indicated an overall passive motivation for oral mucosal screening. This finding agreed with previous studies, which showed that barriers to oral mucosal screening included a lack of a physician recommendation [50], practical barriers (cost and lack of public transport) [51], emotional barriers (avoidance of cancer information and fear) [51], embarrassment, fear of the procedure or report, living in a rural area, and insufficient resources [43]. Our findings should encourage those working in hospital outpatient departments and those providing community-based health services to advocate for BQ users by actively ascertaining whether they have cancer screening barriers and arranging for a physician recommendation, to help BQ users overcome any barriers to cancer screening.

### Limitations

Several limitations affected this study. First, the participants were selected through random sampling from a leading medical center in northern Taiwan. This approach limits the generalizability of the finding to BQ users receiving oral mucosal screening who live in other areas of Taiwan or in other countries. Thus, future studies should recruit BQ users from other areas of Taiwan and from other countries. Second, the participants were assessed through subjective measurements using a set of questionnaires. This approach may have decreased the validity of the results. Thus, future studies should use objective assessment to comprehensively confirm oral health status, anxiety, BDQ, and OHRQoL. Finally, among the variables used in this study, we did not include barriers to health care resulting from the COVID-19 pandemic. Future studies should consider the role of such special events, to examine the correlation between current rates of and motivation for cancer screening.

### Clinical implications

BQ users are at high risk of developing precancerous oral lesions and oral cavity cancer. Improving either oral health, anxiety, or BQD can promote greater OHRQoL in this population. Healthcare providers should educate BQ users to conduct mouth self-examination in order to detect oral mucosal lesions early and facilitate their regular receipt of oral mucosal screening.

## Conclusions

BQ users receiving oral mucosal screening who had greater BQD, a high level of anxiety, annual family income less than NT$500,000, and poor oral health status were more likely to have worse OHRQoL. BQ users with BQD had poorer oral health status, were more likely to have a high level of anxiety, and had worse overall OHRQoL, physical pain, psychological discomfort, physical disability, psychological disability, social disability, and handicap compared to those without BQD.

## Data Availability

The data that support the findings of this study are available from the corresponding author. Restrictions apply to the availability of these data, which were used under license for this study. Data are available from the authors with the permission of the Chang Gung Memorial Hospital Research Program (CMRP).

## Abbreviations

BQ: Betel quid
BQD: Betel quid dependence
BQDS: Betel quid dependence scale
NT$: New Taiwan dollars
OHAT: Oral health assessment tool
OHIP: Oral health impact profile
OHRQoL: Oral-related quality of life
SAI: State anxiety inventory
SD: Standard deviation
